# Supplementary material for: Oxidized Dietary Oil, High in Omega-3 and Omega-6 Polyunsaturated Fatty Acids, Induces Antioxidant Responses in a Human Intestinal HT29 Cell Line
Source: Nutrients. 2022 Dec 15;14(24):5341. doi: 10.3390/nu14245341 (PMC9782097; doi:10.3390/nu14245341)
Supplement: Supplementary file 1 [file nutrients-14-05341-s001.zip › nutrients-2063010-supplementary.pdf]

Table S1. Primer information for quantitative PCR.

Gene	Primer name	Genbank accession no.	(5′-3′)
EF1A	Hsa 25-ef1a-F1	NM_001402.5	GACACGTAGATTCGGGCAAGTCCA
	Hsa 26-ef1a-R1		CCATCTCAGCAGCCTCCTTCTCAA
RPOL2	Has 31-rpol2-F2	NM_000937.4	GCGCAATGAGCAGAACGGCG
	Has 32-rpol2-R2		ACTTCTGCATGGCACGGGGC
GAPDH	Hs33-gapdh-F1	NM_002046.3	ATCCCATCACCATCTTCCAGGAGC
	Hs34-gapdh-R1		AAATGAGCCCCAGCCTTCTCCAT
SOD1	Hsa95 sod1 F2	NM_000454	GGAAGTCGTTTGGCTTGTGG
	Hsa96 sod1 R2		GGGCCTCAGACTACATCCAAG
SOD2	Hsa97 sod2 F1	NM_000636.	ACCACGATCGTTATGCTGAGT
	Hsa98 sod2 R1		TGACTAAGCAACATCAAGAAATGCT
SOD3	Hsa89 sod3 F1	NM_003102	CTCTGAGGTCTCACCTTCGC
	Hsa90 sod3 R1		AAGGATGGTGGGTCTCGGTA
CAT	Hsa101 cat F2	NM_001752	ACTTCTGGAGCCTACGTCCT
	Hsa102 cat R2		TCTGGAATCCCCCGATCACT
XBP1	Hsa 1-XBP1-F1	NM_005080	GCGGAAGCCAAGGGGAATGAAGT
	Hsa 2-XBP1-R1		GCACGTAGTCTGAGTGCTGCGG
ATF6	Hsa 7-atf6-F2	NM_004381	TGGGGTCTGCAGAACAGCACCT
	Hsa -8atf6R2		CCTGCTCCGGGCAACGGAAG
NFKB	Hsa 13-nfkb-F1	NM_03998	GAGGAAGAAAATGGTGGAGTCTGGG
	Hsa 14-nfkb-R1		TCCTCCGAAGCTGGACAAACACA
GPX2	Hsa 19-gpx2-F2	NM_002083	GGGGCTCACTCTGCGCTTCA
	Has 20-gpx2-R2		GCCCTGCCCCGGAACGTATT

Table S2. Relative expression level of genes involved in antioxidant response and ER stress in HT29 cells cultured in increasing concentrations of HHE and HNE for 6 hours. Data (n=4) are means shown with standard error mean. Significant differences are indicated with different letters evaluated with Tukey-HSD (p<0.05).

	Control	HHE				HNE			
		3.1 nM	9.2 nM	27.6 nM	82.9 nM	2.2 nM	6.7 nM	20.2 nM	60.6 nM
SOD1	0.0 ± 0.1	0.1 ± 0.1	0.1 ± 0.1	-0.3 ± 0.2	0.0 ± 0.2	-0.2 ± 0.2	0.0 ± 0.2	-0.2 ± 0.2	-0.3 ± 0.1
SOD2	0.0 ± 0.1 a	-0.4 ± 0.1 a	-0.1 ± 0.1 a	-0.5 ± 0.2 a	-0.4 ± 0.1 a	-0.4 ± 0.1 a	-0.4 ± 0.2 a	-0.4 ± 0.2 a	-1.2 ± 0.1 b
SOD3	0.0 ± 0.3	0.0 ± 0.3	0.3 ± 0.2	-0.3 ± 0.3	0.0 ± 0.1	-0.1 ± 0.3	-0.2 ± 0.2	-0.1 ± 0.4	-0.8 ± 0.3
GPX	0.0 ± 0.1	-0.2 ± 0.1	0.2 ± 0.1	0.2 ± 0.1	0.1 ± 0.1	0.2 ± 0.1	0.2 ± 0.1	0.2 ± 0.2	0.0 ± 0.1
CAT	0.0 ± 0.1	-0.2 ± 0.1	0.0 ± 0.1	-0.3 ± 0.1	-0.2 ± 0.1	-0.2 ± 0.1	-0.2 ± 0.1	-0.1 ± 0.1	-0.3 ± 0.1
ATF6	0.0 ± 0.1 ab	0.0 ± 0.1 ab	0.2 ± 0.1 a	-0.1 ± 0.1 ab	-0.4 ± 0.1 b	-0.1 ± 0.1 ab	0.0 ± 0.1 ab	0.3 ± 0.1 a	-0.3 ± 0.1 b
XBP1	0.0 ± 0.0	-0.3 ± 0.1	0.1 ± 0.0	-0.3 ± 0.1	-0.2 ± 0.1	-0.2 ± 0.1	-0.2 ± 0.1	-0.1 ± 0.1	-0.3 ± 0.1

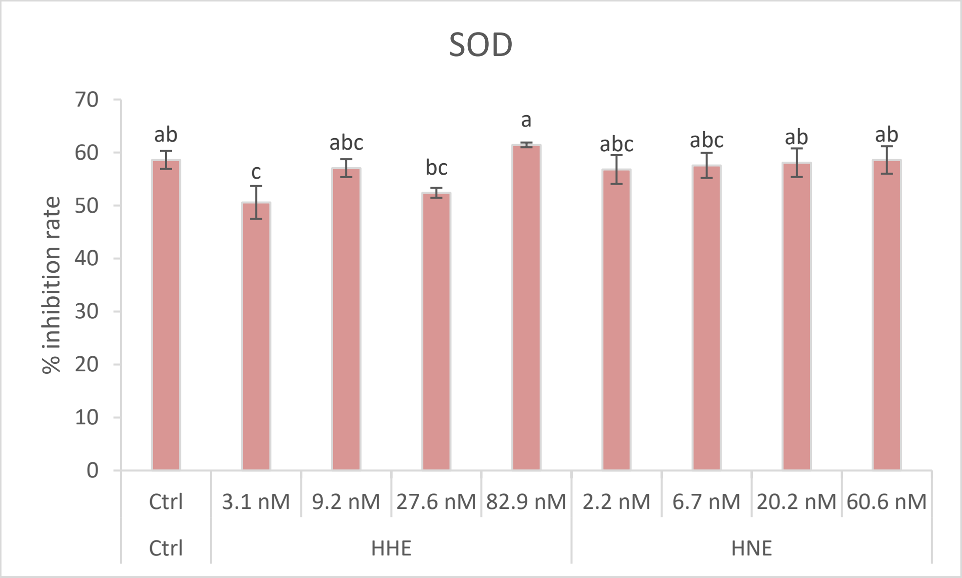

Figure S1. Activity of SOD (% inhibition rate) in HT29 cells exposed to increasing concentrations of HHE and HNE. All groups, except the Control group (Ctrl), were cultured in growth media containing 3.1, 9.2, 27.6 or 82.9 nM HHE or 2.2, 6.7, 20.2 or 60.6 nM HNE for 48 hours. Data (n=4) are means shown with standard error mean. Significant differences are indicated with different letters evaluated with Tukey-HSD ($p<0.05$).
